# The solid tumor microenvironment and related targeting strategies: a concise review

**DOI:** 10.3389/fimmu.2025.1563858

**Published:** 2025-03-26

**Authors:** Yingliang Wang, Huimin Zhou, Shuguang Ju, Xiangjun Dong, Chuansheng Zheng

**Affiliations:** ^1^ Department of Radiology, Union Hospital, Tongji Medical College, Huazhong University of Science and Technology, Wuhan, China; ^2^ Hubei Key Laboratory of Molecular Imaging, Wuhan, China; ^3^ Hubei Provincial Clinical Research Center for Precision Radiology & Interventional Medicine, Wuhan, China; ^4^ Department of Nuclear Medicine, Tongji Hospital, Tongji Medical College, Huazhong University of Science and Technology, Wuhan, China

**Keywords:** tumor microenvironment, classification, characteristic, targeting strategies, perspective

## Abstract

The malignant tumor is a serious disease threatening human life. Increasing studies have confirmed that the tumor microenvironment (TME) is composed of a variety of complex components that precisely regulate the interaction of tumor cells with other components, allowing tumor cells to continue to proliferate, resist apoptosis, evade immune surveillance and clearance, and metastasis. However, the characteristics of each component and their interrelationships remain to be deeply understood. To target TME, it is necessary to deeply understand the role of various components of TME in tumor growth and search for potential therapeutic targets. Herein, we innovatively classify the TME into physical microenvironment (such as oxygen, pH, etc.), mechanical microenvironment (such as extracellular matrix, blood vessels, etc.), metabolic microenvironment (such as glucose, lipids, etc.), inflammatory microenvironment and immune microenvironment. We introduce a concise but comprehensive classification of the TME; depict the characteristics of each component in TME; summarize the existing methods for detecting each component in TME; highlight the current strategies and potential therapeutic targets for TME; discuss current challenges in presenting TME and its clinical applications; and provide our prospect on the future research direction and clinical benefits of TME.

## 1 Introduction

The malignant tumor is a serious disease threatening human life. The World Health Organization reported there were approximately 19.3 million new cancer cases and 10 million cancer deaths worldwide in 2020. The incidence and mortality of cancer are increasing year by year, and it is estimated that by 2040, the global cancer burden will reach 28.4 million people, an increase of 47% over 2020 (1).

Tumor is a heterogeneous disease caused by the accumulation of cell mutations (2). It is a complex heterotypic tissue composed of tumor cells, stromal cells, and extracellular matrix. The extracellular matrix and stromal cells constitute the survival environment of tumor cells, that is, the tumor microenvironment (TME). The concept of TME can be traced back to the close link between inflammation and tumorigenesis and development proposed by Rudolf Virchow in 1863 (3). Since then, many studies have explored the characteristics of TME and the complex relationships between its different components. In 1889, Stephen Paget proposed the hypothesis of “seed and soil”, he believed that metastatic tumor cells represented “seeds” and tissues represented “soil”, and only the formation of a microenvironment suitable for the growth of “seeds” in tissues (that is, soil) could lead to metastasis (4). In 2011, Hanahan and Weinberg described the main characteristics of tumors and strengthened the important role of TME in tumor occurrence, development, and metastasis (5).

The components of TME constitute a complex network that precisely regulates the interaction between tumor cells and other components, which enables tumor cells to proliferate continuously, resist apoptosis, escape immune surveillance and clearance, and metastasize to distant regions (6, 7). There is increasing evidence that malignant tumor is not only an isolated disease but also a disease involving genes, metabolism, inflammation, and immunity (8–10). The theory of TME replaces the theory that the fate of tumor cells is only determined by their genes, and the exploration of TME contributes to the in-depth understanding of tumors. In the early stage, a large number of studies focused on tumor cells, but with the continuous understanding of tumor structure, TME has been proved to play a key role in the process of tumor occurrence, development, metastasis, drug resistance, and the acquisition and maintenance of tumor characteristics depend on the role of TME to varying degrees (11, 12). Therefore, tumor therapy should not only focus on tumor cells but also focus on the remodeling of TME. Tumor cells are heterogeneous and prone to genetic mutations and epigenetic changes, which induce resistance to anti-tumor drugs. The other components of TME have relatively stable gene properties and are more vulnerable to anti-tumor drugs, and are not easy to develop drug resistance (13). At present, therapies targeting TME and simultaneously targeting TME and tumor cells have been emerging, which has become a new trend in tumor treatment (14–16). At the same time, the rapid development of nanotechnology also provides new ideas for the treatment of tumors, such as increasing the targeting and slow-release properties of drugs (17, 18).

To target TME, it is necessary to deeply understand the role of various components of TME in tumor growth and search for potential therapeutic targets. Therefore, this review will present the current understanding of TME and strategies to target TME and open up new ideas for anti-tumor therapy.

## 2 Characteristics of TME

**Figure 1:**
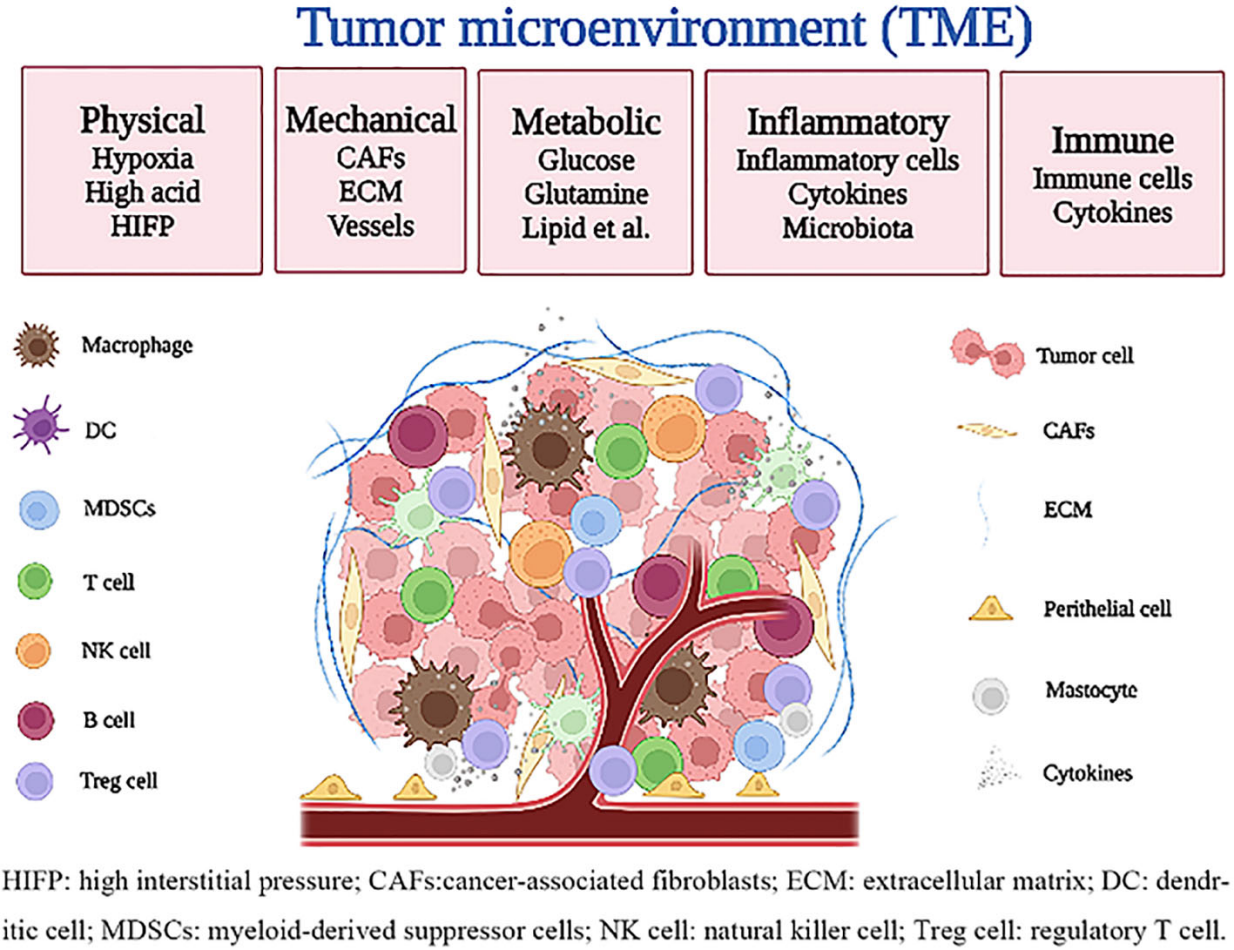
Characteristics of the tumor microenvironment.

Many studies have confirmed the physical microenvironment (such as oxygen, pH, etc.) (19, 20), mechanical microenvironment (such as extracellular matrix, blood vessels, etc.) (21, 22), metabolic microenvironment (such as glucose, lipids, etc.) (23), inflammatory microenvironment (3, 24), and immune microenvironment (10, 25) constitute the TME, which significantly affect the occurrence and development of tumors. Furthermore, these microenvironments have their unique functions but interact with each other (**Figure 1**).

### 2.1 Physical microenvironment

#### 2.1.1 Hypoxia

As early as 1955, hypoxia was recognized as an important marker of solid tumor TME (26). Tumor hypoxic microenvironment refers to the area within the tumor where the oxygen partial pressure is less than 10 mmHg (27). Hypoxia is a state of low oxygen, mainly caused by the imbalance of oxygen supply and consumption in the tumor. On the one hand, rapidly proliferating tumor cells lead to increased oxygen consumption (28); On the other hand, the formation of disordered and leaky non-functional blood vessels induced by hypoxia in tumors significantly decreased oxygen supply (29, 30). In addition, in larger tumors, tumor tissues farther away from blood vessels have poor oxygen supply due to the limited diffusion distance of oxygen (31).

In 2019, three scientists, Kaelin, Ratcliffe, and Semenza, were awarded the Nobel Prize in Physiology or Medicine for their work on how cells sense and adapt to oxygen. Hypoxia leads to the activation of many downstream gene targets, which mainly rely on hypoxia inducible factors (HIFs) mediated signaling pathways to adapt to hypoxic conditions. HIFs consist of constitutionally expressed β subunits located in the nucleus and oxygen-dependent α subunits (HIF-1α, HIF-2α, HIF-3α) located in the cytoplasm, which are stable dependent on hypoxia-inducing factor- prolyl hydroxylases (PHDs) (32). Under normal oxygen conditions, two prolyl residues of the HIF-α subunit are hydroxylated by PHDs, and HIF-α is rapidly degraded (less than 5 minutes) by the ubiquititation-proteasome system (33, 34). However, under hypoxic conditions, the activity of PHDs is inhibited and HIF-α binds to HIF-β subunits to form heterodimers. Subsequently, the heterodimer (HIF-α:HIF-β) migrates to hypoxic response elements (HREs) of the target gene and activates the transcription of multiple genes involved in multiple cellular pathways (35). Of the three subunits of HIF-α, HIF-1α, and HIF-2α are the most studied. Holmquist-Mengelbier and colleagues demonstrated that the difference between the two subunits lies not only in the genes they transcribe but also in the conditions under which they are stable, that is, in the timing of their reactions to oxygen; HIF-1α appears to be most active in the acute phase of hypoxia adaptation, while HIF-2α predominates in the subsequent chronic hypoxia phase (36). In addition, although oxygen-induced signal transduction is mainly mediated by HIF proteins, there are still some signaling pathways that are independent of HIFs for activation. For example, activation of the NF-κB signaling pathway and tumor suppressor p53 can occur in a non-HIF_S_-mediated hypoxia environment (31).

Hypoxia is a common and complex feature of tumor cells and stromal cells in solid tumors. Studies have shown that hypoxia is heterogeneous among different tumor types and different patients with the same tumor type. Moreover, higher hypoxia signals and expression of hypoxia-related genes tend to occur in tumors with high heterogeneity and are associated with poor prognosis and tumor progression (37–39). Oxygen deficiency is present in tumor cells and their microenvironment and subsequently recodes the biological behavior of the tumor in different ways. Under hypoxic conditions, the glycolysis rate is increased by upregulating and/or activating a series of enzymes that promote glycolysis (such as lactate dehydrogenase A, pyruvate kinase M2, etc.) (40) (40); Some acid-releasing transporters (such as monocarboxylate transporter isomer 4, Na^+^/H^+^ exchanger NHE1, etc.) are upregulated by HIF-1α (41, 42), leading to the secretion of various acidic products. Hypoxia promotes the phenotypic transformation of tumor cells to be more aggressive and promotes tumor resistance to apoptosis, dormancy, REDOX adaptation, progression, and metastasis (32, 43–45). Hypoxia activates hypoxia signaling pathways and promotes extensive angiogenesis (46). Hypoxic microenvironments are considered to be the main mechanism leading to tumor resistance to multiple therapies including chemotherapy, radiotherapy, and immunotherapy (28, 31, 47).

#### 2.1.2 Acidity

The acid microenvironment is another important feature of the tumor’s physical microenvironment. In the 1920s, Otto Warburg et al. first described the “Warburg effect”, that is, tumor cells can obtain energy at a disproportionately high rate of anaerobic glycolysis even under aerobic conditions (48). Tumor cells can also obtain energy through glutamine hydrolysis (49). Through these two pathways, large amounts of lactic acid are produced and subsequently excreted outside the tumor cells (i.e., TME). This excess and continuous production of lactic acid leads to acidic TME. In addition, carbonic anhydrase generates additional H^+^ by catalyzing the excessive CO_2_ hydration produced by the pentose phosphate pathway in tumor cells, which also contributes to the formation of acidic TME (50). In addition, the non-functional tumor vascular system formed in hypoxic conditions prevents the effective clearance of hydrogen ions in extracellular mediators. Therefore, the formation of an acidic microenvironment is inseparable from a hypoxic microenvironment and a metabolic microenvironment. Studies have shown that compared with normal tissue cells, tumor cells tend to have higher intracellular pH (7.4 vs. 7.2) but lower extracellular pH (6.5 to 7.0 vs.7.4). This inverted gradient is maintained by the action of various transporters, mainly Na^+^/H^+^ exchangers, H^+^/K^+^-ATPase, Na^+^/HCO_3_^−^ cotransporters, carbonic anhydrase, and monocarboxylate-H^+^ cotransporters (9, 19).

Acidic microenvironment promotes the survival, proliferation, invasion, and anti-apoptosis of tumor cells (50, 51); Promotes tumor metastasis by inducing the expression of specific genes that are different from hypoxia in cells and increasing the extracellular levels of factors such as proteolytic enzymes (50, 52); Promotes angiogenesis by inducing the secretion of vascular endothelial growth factor (VEGF) and IL-8 (NF-kB pathway) (53, 54); Induces inflammation and immune escape, such as inducing the polarization of macrophages towards M2 phenotype, activating neutrophils and promoting inflammation, inhibiting the activation of dendritic cells, and inhibiting the cytotoxic activity of infiltrating lymphocytes (TIL) (55, 56); Induces resistance to anti-tumor therapies such as chemotherapy and radiotherapy in a variety of ways, such as activation of P-glycoprotein and p38 MAPK pathways (15, 28, 57).

#### 2.1.3 High interstitial pressure

High interstitial pressure (HIFP) is an important marker of malignant tumor growth. In normal microcirculation, fluid seeps from the arterial segment of the capillaries and returns to the blood circulation through the lymphatic vessels or venules within the extracellular matrix (ECM), thereby maintaining a low level of interstitial pressure (0 to 3 mm Hg) (58, 59). In tumors, however, several mechanisms break this normal cycle: the rapid proliferation of tumor cells within a limited space; Leakage of new blood vessels to supply the nutrients and oxygen needed for the rapid proliferation of tumor cells; The structure and function of blood vessels and lymphatic vessels within the tumor are abnormal, which cannot drain the metabolites produced by tumor cell proliferation; Contraction and fibrosis of ECM (60). As a result, a large amount of fluid accumulates in the tumor stroma, resulting in stromal pressure of 10 to 40 mm Hg, which can be as high as 100 mm Hg in malignant melanoma (61).

HIFP limits the transfer of oxygen and nutrients, exacerbating hypoxia and acidity; In addition, HIFP is considered to be a major obstacle to the delivery of antitumor drugs to their target when administered either systemic (intravenous or oral) or locally (intracavitary or intratumoral) (62). Many studies have confirmed that HIFP can change the biological behavior of tumors and is a predictive parameter of tumor progression (60, 63).

### 2.2 Mechanical microenvironment

#### 2.2.1 Tumor-associated fibroblasts

Fibroblasts are resting in normal tissues, but they can be activated by growth factors secreted by tumor cells and differentiate into myofibroblasts. This activation is permanent and resistant to apoptosis and is often referred to as tumor-associated fibroblasts (CAFs) (64, 65). CAFs express certain specific biological markers, including α-smooth muscle actin (α-SMA), fibroblast specific protein-1 (FSP1), and fibroblast activating protein (FAP) (66).

CAFs play an important role in tumor growth and metastasis. By unregulating Notch and p53 signaling pathways and secreting matrix metalloproteinases (MMPs), growth factors, and cytokines, they stimulate angiogenesis, inflammatory response, cell proliferation, and invasion (67, 68). CAFs affect the toughness of ECM and enhance cell invasiveness by inducing epithelial-mesenchymal transformation (EMT), which is conducive to metastasis and dissemination (69). CAFs express intercellular adhesion molecule 1 (ICAM1) and programmed cell death protein 1 ligand 1/2 (PDL1/2), inducing T cell depletion and immune tolerance (70). CAFs can recruit myeloid-derived immunosuppressive cells (MDSCs) and regulatory T cells (Tregs) by up-regulating the expression of chemokines CCL22 and CCL28, and further enhancing immunosuppressive TME (71). Inhibitory immune cells, in turn, can regulate CAFs, thereby exacerbating connective tissue formation in TME (72).

A large number of studies have also shown that CAFs can induce drug resistance, including chemotherapy drugs, targeted drugs, immunosuppressants, and other drugs. The main resistance mechanisms include the delivery of exosomes that stimulate tumor cell survival; Promote EMT of tumor cells and reduce the expression of transporters that absorb the corresponding drugs; Chemotherapeutic drugs are eliminated to reduce the dose of chemotherapy drugs within the tumor (13).

#### 2.2.2 Extracellular matrix

ECM is a complex structure composed of proteins, proteoglycans, and other molecules. The main proteins in ECM are fibrin and proteoglycan, which promote the formation of tissue hardness and basement membrane, and the latter acts as a barrier between tumor cells and stroma. In addition to protein components, cytokines, growth factors, hormones secreted by stromal cells and tumor cells, as well as physical and chemical parameters such as pH, oxygen, and interstitial pressure, are also components of ECM (73). In tumor tissues, the remodeling of ECM may result from the action of extracellular matrix regulatory enzymes, thus affecting the biological behavior of tumors. In addition, by releasing some growth factors, the downstream signaling pathways (such as TGF-β/Smad, PI3K/Akt, MAPK, etc.) are activated to promote the occurrence and progression of tumors (22, 74). Therefore, ECM not only provides physical structural support for various tissues but also is responsible for the signal connections between cells and cells and stroma.

Integrin is highly expressed on the surface of tumor cells and vascular endothelial cells, mediating intercellular and cell-ECM interactions (75). As a receptor for adhesion molecules and various proteins, integrins affect the adhesion, migration, and survival of tumor cells. Some studies have found that integrin is expressed on the surface of new blood vessels, thereby regulating tumor angiogenesis. Integrins can also affect the expression of multiple stroma-degrading enzymes, thus affecting the invasion of tumor cells (76, 77). HIF-1 can promote the production of lysine oxidase to enhance the integrin signaling pathway and increase the hardness of the tumor matrix (78). HIF-1 can activate the transcription of genes encoding degrading proteases or reshape ECM in primary tissue and distant metastatic sites (79). HIFs can also regulate cell proliferation and survival by binding to hypoxic response elements in genes (79).

ECM remodeling increases the hardness of tumors, which is mainly caused by the increase of ECM components, especially collagen and hyaluronic acid (80) (80). ECM hardening can lead to intracellular contraction, which in turn increases the hardness of the actin cytoskeleton, which is conducive to tumor migration (81) (81). Stromal sclerosis can also activate TGF-β signaling and EMT, resulting in the transformation of tumor cells to a more aggressive phenotype (22).

MMP plays an important role in the regulation of ECM remodeling and can degrade mostly various proteins in ECM, promoting tumor cell proliferation and EMT. For example, it resists cell apoptosis by hydrolyzing certain pro-apoptotic factors (8); By degrading ECM and releasing related angiogenic factors and inhibitors, they jointly regulate the formation of tumor blood vessels and play an important role in the process of lymphangiogenesis and lymphatic metastasis (82, 83).

#### 2.2.3 Vascular structure

Tumors are rich in blood vessels and lymphatic vessels that supply nutrients, oxygen, and waste clearance. Tumor angiogenesis originates from two different biological processes: angiogenesis by “budding” on the basis of existing blood vessels and angiogenesis by recruitment of circulating endothelial progenitor cells to form new blood vessels (84). The process of angiogenesis is regulated by a variety of factors and signaling pathways, such as vascular endothelial growth factor A (VEGF-A), fibroblast growth factor (FGF), platelet-derived growth factor (PDGF), angiogenin and other pro-angiogenic factors, which can promote the proliferation, migration, differentiation, and maturation of endothelial cells and pericytes (85). However, angiogenic inhibitors such as endostatin and angiostatin hinder the generation of blood vessels (86). As the homeostasis of angiogenesis-promoting factors and inhibitors is unbalanced within the tumor, new blood vessels are constantly generated, and the biological characteristics of new blood vessels are also changed, resulting in the disorder, irregularity, and leakage of the generated blood vessels, and the loss of normal function (87).

Distinct from classical angiogenesis, vasculogenic mimicry (VM) provides a blood supply for tumor cells independent of endothelial cells (88). In recent years, VM has been reported in many malignant tumors, such as melanoma, hepatocellular carcinoma, breast cancer et al. (88–90), and is associated with tumor progression, invasion, and metastasis (91). The mechanism of VM formation has not been fully elucidated, and the potential mechanisms reported in current literature include EMT, hypoxia, cancer stem cells, vascular endothelial-cadherin, MMP, and so on (89, 92).

Vasculogenic cancer-associated fibroblasts (vCAFs) are subtype of CAFs, which have been reported to induce tumor angiogenesis through various pathways (93). The vCAFs can produce several angiogenesis factors, such as VEGF, FGF, PDGF, and osteopontin to promote the vessel formation by recruiting myeloid cells and accelerate angiogenesis by attracting vascular endothelial cells and recruiting monocytes (94, 95). The vCAFs can also increase the formation of VM, and increase the contact between tumor cells and vCAFs via the Notch2-Jagged1 pathway (96). Besides, vCAFs can increase IL-6 and IL-8 secretion and promote angiogenesis in intrahepatic cholangiocarcinoma and colorectal Cancer (97, 98).

HIFs regulate angiogenesis by inducing the transcription of VEGF and other vascular growth factors under hypoxic conditions (99). Studies have reported that long-chain non-coding RNAs (lncRNA), such as lncRNA F630028O10Rik and lncRNA H19, can promote angiogenesis by up-regulating the expression of VEGFA and other pro-angiogenic factors (100, 101). Other studies have confirmed the correlation between bone marrow mesenchymal stem cells (MSCs), mast cells, and tumor-associated macrophages (TAMs) and angiogenesis (102).

The lymphatic vessels within the tumor are composed of a single layer of lymphatic endothelial cells with a discontinuous basement membrane and a lack of peripheral cell or smooth muscle cell support (103). Lymphangiogenesis is regulated by a variety of growth factors, of which VEGF-C and VEGF-D, which recognize the vascular endothelial growth factor receptor-3 (VEGFR-3) on the surface of lymphatic endothelial cells, are the most important (104). In addition, VEGF-A and angiopoietin can also promote lymphangiogenesis, while endostatin can inhibit lymphangiogenesis (105–107).

The disorganized nature of the tumor vascular system reduces the penetration of anti-tumor drugs, but also hinders the entry of immune cells and promotes immune escape, which is related to tumor recurrence (31, 99, 108). The generated lymphatic vessels are also nonfunctional, and together with nonfunctional blood vessels, they cause HIFP, which also hampers drug delivery. In addition, a large number of studies have shown that lymph node metastasis and distal metastasis of tumors are closely related to lymphangiogenesis (106).

### 2.3 Metabolic microenvironment

#### 2.3.1 Glucose

Different from normal cells, which mainly rely on oxidative phosphorylation to obtain energy, tumor cells are more inclined to shift to a metabolic direction characterized by glycolysis even under aerobic conditions, which is also called the “Warburg effect” or aerobic glycolysis (48). The mammalian target of the rapamycin (mTOR) pathway is a key signaling pathway that regulates metabolic processes. It affects several processes in the glycolysis pathway by regulating the expression of key transcription factors such as HIF-1. The expression of HIF-1α is dependent on mTORC1 and mTORC2, both of which play important roles in glucose uptake and glycolytic metabolism of tumor cells (109, 110).

As mentioned above, tumor metabolism produces a large number of acidic products, inducing the formation of an acidic TME. For many years, lactic acid was simply considered a waste product of tumor metabolism. However, there is evidence that it can reprogram tumor cells and stromal cells in TME. It promotes the polarization of macrophages towards pro-tumor and pro-inflammatory (M2-like) phenotypes; Stimulate angiogenesis, local invasion, and distant metastasis of tumor cells; The production of acidic microenvironment is also a key factor in the immune escape of tumor cells (49, 111). In addition, the interaction between tumor metabolism and the acidic microenvironment is bidirectional, as lower pH inhibits enzymes involved in glycolysis (such as phosphofructokinase-1) and may inhibit tumor cell proliferation and survival (112). Other studies have shown that this process also involves inhibition of the expression of MCT4 (113), stimulation of the mitochondrial metabolism (114), and shifting glucose consumption from lactic acid production to the pentose phosphate pathway (115). To avoid the inhibitory effect of intracellular acidification on tumor cell metabolism, tumor cells expel intracellular acidic products through a series of transporters (as described above in the acidic microenvironment section).

#### 2.3.2 Glutamine

Glutamine is also an important substrate for tumor metabolism. When glucose is deficient, glutamine metabolism dominates, and glutamine supplements energy, carbon, and nitrogen for tumor cells and stromal cells (116, 117). Tumor cells absorb proteins, which in turn can be degraded into glutamine, providing the tumor cells with substances necessary for growth through RAS-activated macropinocytosis (116). Studies have shown that the acidic TME catalyzes the conversion of glutamine to glutamate by increasing the expression of glutamine transporter ACT2 and glutaminase-1 (113). Many other studies have also investigated tumor glutamine metabolism (118, 119). For example, driven by the activation of prolinyl-4-hydroxylase regulated by alpha-ketoglutaric acid, consumption of pyruvate in TME by breast cancer cells leads to hydroxylation of collagen, thereby inducing ECM remodeling (120).

#### 2.3.3 Lipids

Lipids are part of the synthesis of tumor cell membranes, post-translational modification of proteins, and energy sources of tumor cells (121, 122). Fatty acid oxidation was significantly increased in tumor cells living in an acidic TME (123). The acidic environment affects the survival of tumor cells by upregulating a transcription factor (sterol regulatory element binding protein 2) that plays a key role in cholesterol synthesis (124). High levels of cholesterol can be seen in TME, which is positively correlated with CD8^+^ T cell failure (8). In a mouse melanoma model, cholesterol increases endoplasmic reticulum stress, activates X-box binding protein 1, and up-regulates PD-1 expression on CD8^+^ T cells (125). Studies have reported that fatty acid binding protein 4 promotes the dissolution, transport, and metabolism of fatty acids, and its expression in adipocytes and metastatic retinal cells is significantly up-regulated (126). In addition, it has been found that acidic TME can induce lipid droplet accumulation (112, 127).

Hypoxia, metabolic abnormalities, endoplasmic reticulum stress, and activation of proto-oncogenes can induce massive production of reactive oxygen species (ROS) in tumor cells (128), which is closely related to tumorigenesis, progression, immunity, and TME remodeling (129). As the tumor progresses, it develops a mature ROS clearance system (such as increasing the transcription of glutathione), so that the ROS in the tumor cells reaches a certain balance, and breaking this balance, that is, clearing or increasing ROS will affect the survival of tumor cells (130). ROS is required for the stabilization of HIFs under hypoxic conditions, which can further induce autophagy and enhance the malignant phenotype of tumors (131). Locally elevated ROS in TME can affect the growth of tumor cells and the activity of immune cells (132, 133). For example, increased ROS can increase the immunosuppressive function of inhibitory T cells (Tregs) and resistance to PD-1/L1 therapy (134).

#### 2.3.4 Intestinal microbes

The liver is closely connected to the gut through the portal vein system, and the liver is exposed to the gut microbiota and corresponding microbial molecules. In a mouse liver cancer model, intestinal flora, lipopolysaccharide, and TLR-4 promote the progression of liver cancer (135, 136).

The gut flora regulates the production of bile acids. In liver inflammation, the down-regulation of farnisoid X receptor (FXR) signaling can reduce the function of hepatic bile acid transporters, and the subsequent increase of bile acids and continuous inflammation can promote the occurrence of liver cancer (137). The study found that mice lacking FXR showed impaired bile acid homeostasis, which could spontaneously develop liver cancer. Researchers used cholestyramine to reduce bile acid levels, resulting in a reduction in liver tumor burden (138). Gut microbes can regulate the anti-tumor immune response by regulating innate and adaptive immunity in TME, and the presence of certain microbiomes or the imbalance of multiple microbiomes is related to tumor response to immune checkpoint inhibitors (139, 140). In a mouse model of liver cancer, antibiotic-induced clearance of intestinal symbiotic bacteria inhibits liver tumor growth, mediated primarily by increasing liver natural killer T (NKT) cells, which are regulated by chemokine 16 (CXCL16) secreted by hepatic sinus endothelial cells. However, the expression of CXCL16 is controlled by intestinal microbiome-mediated bile acid circulation (141).

### 2.4 Inflammatory microenvironment

Since Rudolf Virchow proposed the close relationship between inflammation and tumor in 1863, more and more studies have confirmed that chronic inflammation is an important marker of tumor occurrence and progression (3). Different types of inflammation have different effects on tumors: oncolytic inflammation plays an anti-tumor role, while chronic inflammation promotes the occurrence, development, and metastasis of tumors (142). Various cells in TME mediate inflammation by secreting cytokines and chemokines and other inflammatory mediators (143). Pro-inflammatory cytokines are beneficial to the EMT process and angiogenesis. Anti-inflammatory cytokines are involved in immune escape and promote tumor progression (24). Tumor-associated macrophages (TAMs), CAFs, natural killer cells (NK cells), mast cells, and MDSCs play an important role in the tumor inflammatory microenvironment (142). TAMs represent a major group of inflammatory cells that secretes a variety of chemokine components, such as CCL2 and CXCL8, and maintain tumor proliferation and immunosuppressive phenotype by inducing TAMs to convert from M1 polarity to M2 polarity (144). It has been found that CAFs play an important role in the activation of NF-κB signal, the production of pro-inflammatory factors, and the up-regulation of pro-inflammatory gene expression (142, 145). Inflammatory cytokines (such as TNF-α and IL-12) can increase the anti-tumor efficacy of NK cells (142). Mast cells can amplify the inflammatory response through the SCF/c-kit signaling pathway. Mast cells assist in the inhibitory function of MDSCs by deploying them to tumor sites via the IL-17 pathway and stimulating IL-17 expression in MDSCs. Mast cells induce Tregs infiltration and induce IL-9 production, and IL-9 promotes the tumor-promoting effect of mast cells (102, 146).

The relationship between microbiota and chronic inflammation is gradually being recognized. Many symbiotic bacteria and their metabolites exist in the gastrointestinal tract, and the formation of many digestive tract tumors is thought to be related to microbe-induced inflammation, such as liver cancer, stomach cancer, and colon cancer (147–149). Tumors affected by intestinal microbiota − inflammation is not limited to the gastrointestinal tract, but can also occur in other areas. When physical or chemical stimulation, antibiotic use, stress, and other reasons cause intestinal mucosal destruction, flora shift/imbalance, and inflammatory substances, through various signaling pathways (such as TLRs) to stimulate the body to produce various inflammatory mediators (such as IL-6 and TGF-β, etc.), at the same time, various immune cells (such as Tregs and MDSCs) can be recruited into TME. Induce inflammation at the tumor site, promote tumor formation and development, and immune escape (149, 150).

### 2.5 Immune microenvironment

**Figure 2:**
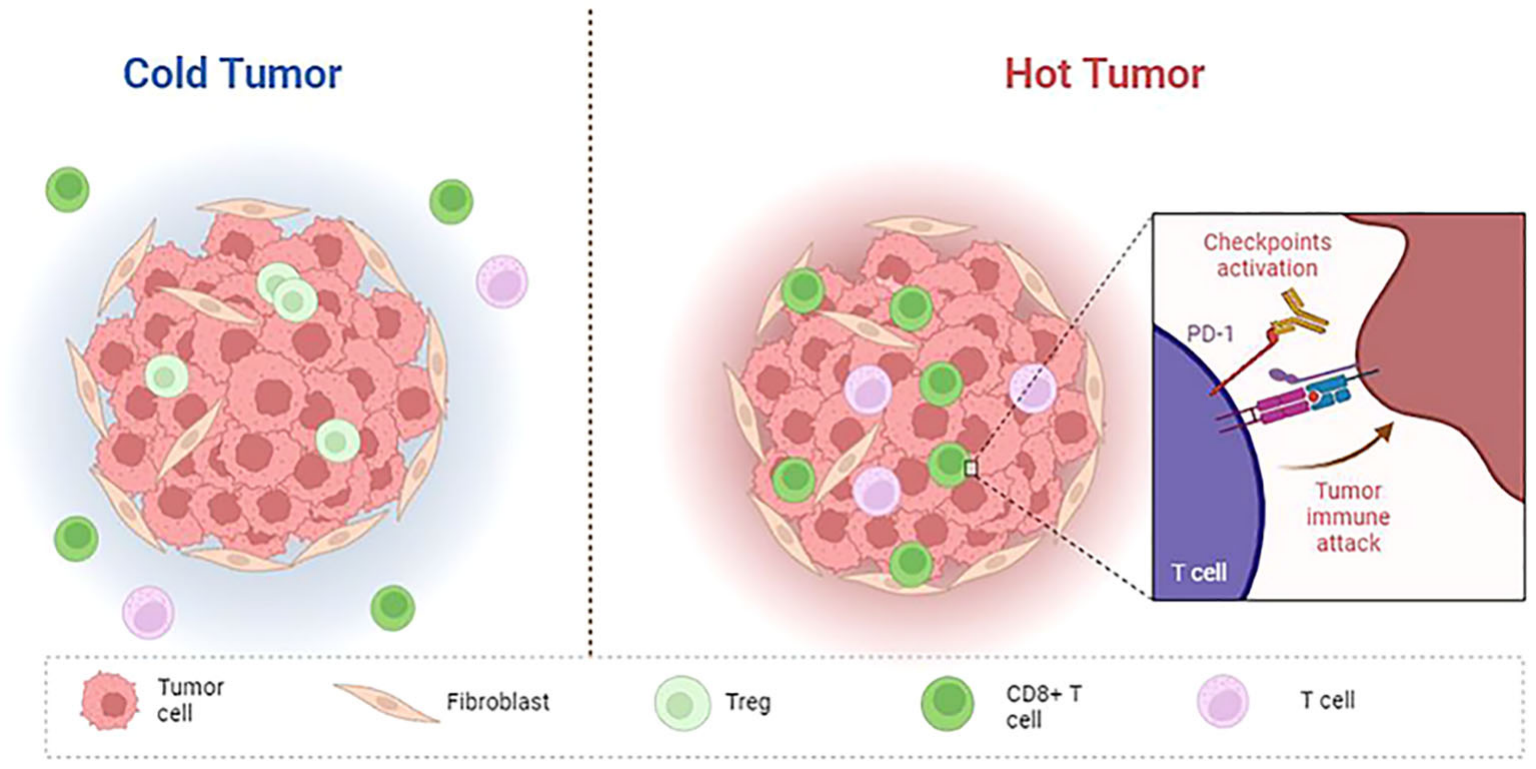
Features of cold and hot tumor. Cold tumor: non-inflammatory, no T cell infiltration within the tumor and at the tumor border; Hot tumor: inflammatory, high levels of T cell and cytotoxic T cell infiltration within the tumor, immune checkpoint (PD-1, CTLA4, LAG3, TIM3) activation.

With the continuous study of tumor immunity, some researchers have proposed several immune subtypes of tumors based on the histological characteristics of tumors (**Figure 2**). Hegde et al. classified it into inflammatory type and non-inflammatory type (immune excluded type and immune desert type). The former is characterized by a large number of functional lymphocytes that can secrete IFN-γ in the tumor, while the latter is characterized by low infiltrating lymphocytes in the tumor (151). Later, Galon et al. further proposed four immune subtypes: Cold tumors (no lymphocyte infiltration within the tumor and at the tumor border), immune excluded tumors (little lymphocyte infiltration at the tumor border), immunosuppressive tumors (little T cell and cytotoxic T cell infiltration within the tumor), and hot tumors (high levels of T cell and cytotoxic T cell infiltration within the tumor) (152). The infiltrated location of immune cells in TME, that is, located at the core or edge of the tumor or near the lymphatic organs or lymph nodes, is closely related to the prognosis of the tumor (153). Hot tumors respond best to immune checkpoint inhibitors, so the researchers advocate a therapeutic strategy that converts “cold tumors” into “hot tumors.” For hot tumors, the key is to remove the inhibition of cytotoxic T cells. For cold tumors, it is necessary to induce the production and activation of tumor-associated T cells (154). In 2018, Thorsson et al. (10) proposed another new tumor immune subtype classification based on extensive immunogenomic analysis of more than 10,000 tumors: wound-healing, IFN-γ-dominant, inflammatory, lymphocyte depleted, immunologically Quiet, and TGF-β dominant. These six stable and replicable immune subtypes are found to be included in almost all human malignancies and are strongly associated with prognosis. TGF-β, a type of cytokines secreted by immune cells and stromal cells, plays an important role in the occurrence and progression of tumors. Its functions were complex in different stages of the tumor. Several studies have shown that TGF-β was a tumor suppressor at an early stage of the tumor by inhibiting the proliferation of immunosuppressive myeloid cells, while it serves as a promoter at the advanced stage of the tumor by suppressing the antitumor immunity, and an increased expression of TGF-β in the TME is thought to be associated with the tumor neovascularization (155).

The components of tumor cells and TME (both cellular and non-cellular components) are involved in shaping the tumor immune microenvironment. The main mechanisms by which tumor cells evade immune response include infiltration of Tregs, damaged antigen presentation, production of multiple immunosuppressive mediators, and differentiation of M2-type macrophages (156). TAMs are mainly produced by mononuclear cells of bone marrow origin or residual macrophages in tissues. TAMs can be differentiated into M1-type macrophages and M2-type macrophages, the former can promote Th1-type immune response and produce abundant pro-inflammatory cytokines, while the latter mainly plays the roles of anti-inflammatory, anti-apoptotic, and induce immune escape (157). In most tumors, infiltrating macrophages are dominated by M2 types, which secrete anti-inflammatory molecules (such as IL-10 and TGF-β) to help shape immunosuppressive TME (158). TAMs resist the infiltration of CD8^+^ T lymphocytes at the tumor site, resulting in the non-responsiveness of anti-PD-1 therapy. The increased expression of immune checkpoint ligand levels in TAMs (such as PD-L1 and PD-L2, etc.) can directly inhibit the activity of T cells; TAMs can recruit Tregs, resulting in the inhibition of T cell activity (159). TAMs can produce a variety of cytokines such as IL-10 and TGF-β in TME, weaken the activity of effector T cells, and inhibit DCs maturation (160). DCs rely on major histocompatibility complex (MHC) molecules to present antigens to T cells and recruit other immune cells by secreting pro-inflammatory cytokines and chemokines. Anti-inflammatory factors in TME interfere with the normal differentiation of monocytes into DCs and promote the production of immature regulatory DCs(RegDCs), which can inhibit the function of T cells by secreting TGF-β and prostaglandin E2(PGE2) (161). Bone marrow-derived suppressor cells (MDSCs), a kind of cell rich in TME, play important roles in angiogenesis, immune evasion, and tumor progression. Firstly, MDSCs can inhibit the activation and promote the apoptosis of T cells; Secondly, MDSCs can participate in the activation of TAMs, which promote the conversion of M1-TAM to M2-TAM; Thirdly, MDSCs can express and secrete VEGF, IL-1/2/10, TGF-β, PDL 1/2, inducible nitric oxide synthase (iNOS), indoleamine 2,3-dioxygenase (IDO), and arginase-1 (ARG-1), which could recruit immunosuppressive cells and induce tumor immune evasion (162, 163).

The low expression of signaling molecules in TME (such as MHC Class I chain-related proteins A and B driving activation receptors) on NK cells inhibited its anti-tumor effect; NK cells and CTL cells play a key role in eliminating malignant tumors, and the metabolism of these two cells is interdependent with tumor cells, resulting in metabolic competition, nutritional restriction, and immunosuppression (72). In addition to IL-10 and TGF-β mentioned above, another immunomodulator that has been widely studied in tumor therapy is indoleamine-2, 3-dioxygenase 1 (IDO1) enzyme, which and its metabolites can inhibit T cell function, induce DCs apoptosis, and support Tregs phenotypic transformation. It is also associated with shorter survival and immunotherapy resistance in many solid tumors (164).

Other non-tumor cells and ECMs in the TME also help shape the immunosuppressive microenvironment. To induce the depletion and impotence of effector T cells, the peripheral cells, CAFs and MDSCs, and T cells can be induced to express immunoglobulin and PDL1 on their surfaces, and secret related factors such as IDO and TGF-β (72). It has been suggested that stroma may be a barrier to antigen presentation and immune recognition (165).

## 3 Methods for detecting TME

There are many methods to measure physical and chemical indexes in TME, but they are mostly used in scientific research (**Table 1**). The following methods can be used to detect hypoxia: Oxygen electrode is an invasive method for directly measuring tissue oxygen partial pressure, and is the gold standard for detecting oxygen content in the tissue (166); It can be measured by implantable paramagnetic crystals (whose interaction with oxygen is measured by electron paramagnetic resonance) or by oxygen-sensitive fluorescent probes attached to fiber optic cables (OxyLite) (9). Positron emission tomography (PET) is a metabolic and functional imaging technique used in the clinic to evaluate the oxygen content of tumors. Different tracers (mainly 2-nitroimidazole compounds) can enter cells by passive diffusion, where they can be retained for imaging. Currently, tracer probes widely used for hypoxia imaging include ^18^F-FDG, ^18^F-FMISO, ^18^F-FAZA, ^18^F-EF5, and ^131^I-IAZGP. PET has high sensitivity and specificity to hypoxia, but its spatial resolution is relatively low (167). The BOLD-MRI technique (which presents different signals with different oxygenated and deoxyhemoglobin content in the tumor) can also measure the oxygen content in the tumor noninvasively (168). Immunofluorescence and immunohistochemical methods can measure oxygen content in tissue indirectly by evaluating the expression of hypoxic-related proteins, commonly used related proteins include exogenous hypoxic markers (pimonidazole, EF-5) and endogenous cell markers (HIF-1, VEGF, CA9, GLUT-1/3) (46). The following methods can be used for the detection of the extracellular acidic environment: Direct measurement through the invasive insertion of electrodes is the gold standard (169); Non-invasive methods for indirect pH detection using endogenous and exogenous compounds, such as magnetic resonance spectroscopy (MRS), MRI, and PET (170, 171); SNARF and BCECF fluorescent probes are also commonly used in the market. After the probes enter the tumor, the probes could present different fluorescence signals at different pH in the tissues, reflecting the difference in tissue pH (169, 172). Tumor interstitial pressure can be measured by embedding capillary electrodes with a semi-permeable membrane into the tumor (173).

**Table 1:** Methods for detecting TME.

Parameters	Detecting methods
Hypoxia	Oxygen electrode; PET(^18^F-FDG, ^18^F-FMISO, ^18^F-FAZA, ^18^F-EF5, and ^131^I-IAZGP); BOLD-MRI; IF and IHC of hypoxic markers (pimonidazole, EF-5) and endogenous cell markers (HIF-1, VEGF, CA9, GLUT-1/3)
Acidity	pH electrodes; MRS; MRI; PET; SNARF and BCECF fluorescent probes
Interstitial pressure	Embedding capillary electrodes
Metabolites	MS; NMR; Isotope tracer techniques
Others	Single-cell sequencing; multi-label immunofluorescence; multi-molecular imaging; transcriptome sequencing; mass spectrometry flow cytometry

*PET*, Positron emission tomography; *Bold-MRI*, Blood oxygen level dependent-Magnetic Resonance Imaging; *IF*, Immunofluorescence; *IHC*, Immunohistochemistry; *MRS*, magnetic resonance spectroscopy; *MS*, mass spectrometry; *NMR*, Nuclear magnetic resonance.

The measurement of metabolites in tumors mainly includes mass spectrometry (MS) and nuclear magnetic resonance (NMR) (9) (9). When measured, tumor samples need to be rapidly frozen, ground into powder, extracted, and the extracted liquid analyzed. Although they can provide valuable information about metabolites, these methods do not allow for real-time microenvironmental changes and metabolite concentrations may change rapidly after cutting off the tumor’s blood supply. In addition, isotope tracer techniques have been used for several years to detect the metabolic status of certain substances in TME, such as ^13^C-glucose *in vivo*. In conclusion, it is still necessary to find multiple accurate methods to evaluate tumor metabolism (174).

Other techniques for measuring the TME include single-cell sequencing, multi-label immunofluorescence, multi-molecular imaging, transcriptome sequencing, and mass spectrometry flow cytometry (175–179). These techniques help to evaluate the information of various components in TME, which is of great significance for the prognosis assessment of tumors and the search for therapeutic targets. However, at present, these technologies also have some shortcomings, such as the research is mostly in the preclinical stage and it is difficult to dynamically observe the tumor microenvironment.

## 4 Strategies for targeting the TME

### 4.1 Targeting the physical microenvironment

#### 4.1.1 Targeting hypoxia

Hypoxia is a state of low oxygen, and the most direct way to solve this problem is to increase the level of oxygen in the tumor through oxygen delivery or oxygen production. Hyperbaric oxygen therapy refers to the use of high pressure to deliver sufficient oxygen to the blood and tumor. However, this type of oxygen delivery, due to its non-tumor specificity, may cause serious side effects to normal tissues, such as barotrauma and seizures due to high oxygen toxicity (180). With the development of nanotechnology, many nanoparticle platforms designed to increase tumor oxygenation are also in preclinical development, including agents that deliver oxygen to the tumor site and produce oxygen *in situ* at the tumor site and have demonstrated their stability, biocompatibility, effective oxygen-carrying, and tumor targeting (181). The methods of carrying oxygen to the tumor site include oxygen carrying of red blood cells and hemoglobin (182, 183), perfluorocarbon oxygen carrying (184), and metal-organic framework structures (such as zirconia-based metal-organic framework (UiO-66), etc.) (185); Methods of *in situ* oxygen production at tumor sites include: Catalase catalyzes hydrogen peroxide oxygen production in tumors (186), nano-enzyme (such as MnO_2_, MnFe_2_O_4_, cerium oxide, etc.) (187), photoinduced oxygen production (that is, under the irradiation of an external light source of a specific wavelength, nanomaterials produce oxygen by catalyzing water or hydrogen peroxide degradation) (188) and metal peroxides by chemical reactions (189).

Stimuli-responsive nanoplatforms have also been considered as a promising and effective targeting strategy against tumors, as these nanoplatforms maintain their stealth feature under normal conditions, but upon homing in on cancerous conditions, are responsive and release their cargoes. Moreover, functionalized nanoparticles can also be activated by external stimuli including magnetic field, light, and ultrasound, to realize efficient tumor accumulation and controlled drug release in a temporal and spatial-specific fashion (190). And stimuli-responsive nanoplatforms-triggered oxygen release has also been reported in literature (191–193). Dual-targeting strategies, which designing nanoparticles that only activates in the presence of both triggers, seemed to achieve higher specificity and reduce off-target effects for diseased tissues. Many dual-responsive tumor-targeting nanoparticles, such as ROS and GSH dual-responsive tumor-targeting strategy, GSH and hypoxia dual-responsive tumor-targeting strategy et al, have been designed in preclinical studies and showed satisfactory anti-tumor effects and few side effects (194–196). However, careful design and validation are essential to address the challenges associated with these advanced systems.

Hypoxic-activated prodrugs (HAPs) are bioreductive agents that are selectively activated under hypoxic conditions to precisely target hypoxic areas within tumors. Representative drugs include Tirazamine, evofosfamide (TH-302), etc. A large number of preclinical and clinical studies have shown that no matter the single drug application or combination application, no matter local administration or systemic administration, it has demonstrated a satisfactory anti-tumor effect (181, 197).

**Figure 3:**
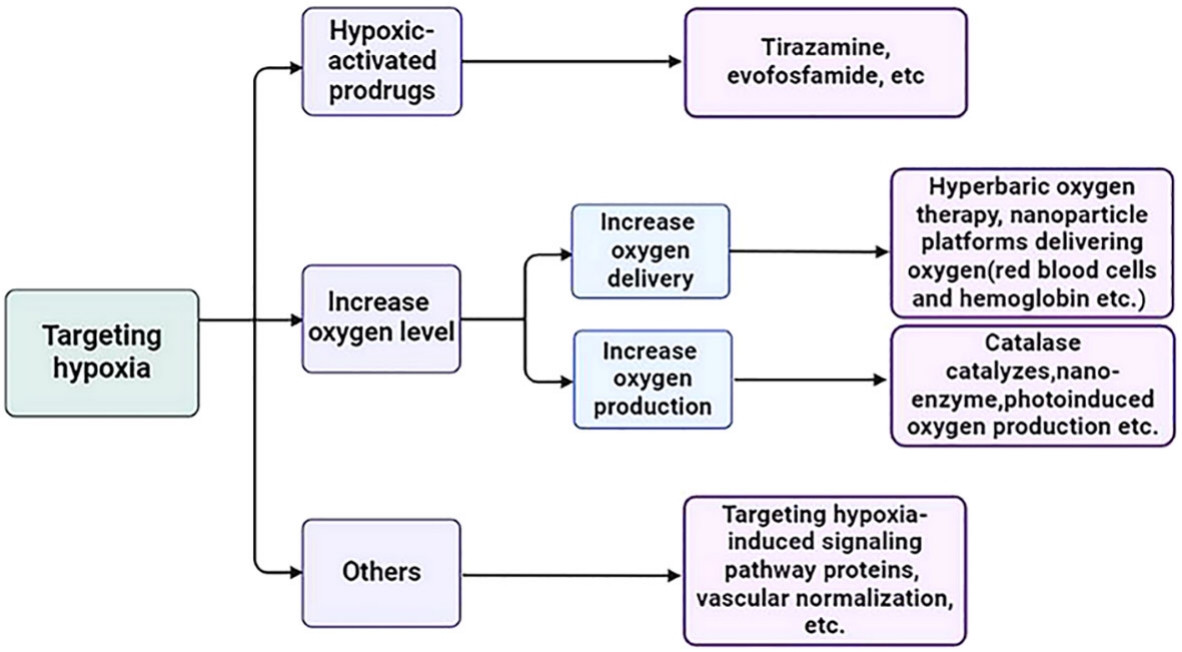
Strategies for targeting hypoxia in TME.

Other measures to target hypoxia include targeting hypoxia-induced signaling pathway proteins (such as HIF-PHD-pVHL pathway proteins and mTOR pathway proteins, etc.) and increasing oxygen supply through vascular normalization (such as the use of antiangiogenic drugs, etc.) (198–200) (**Figure 3**).

#### 4.1.2 Targeting acidity

**Figure 4:**
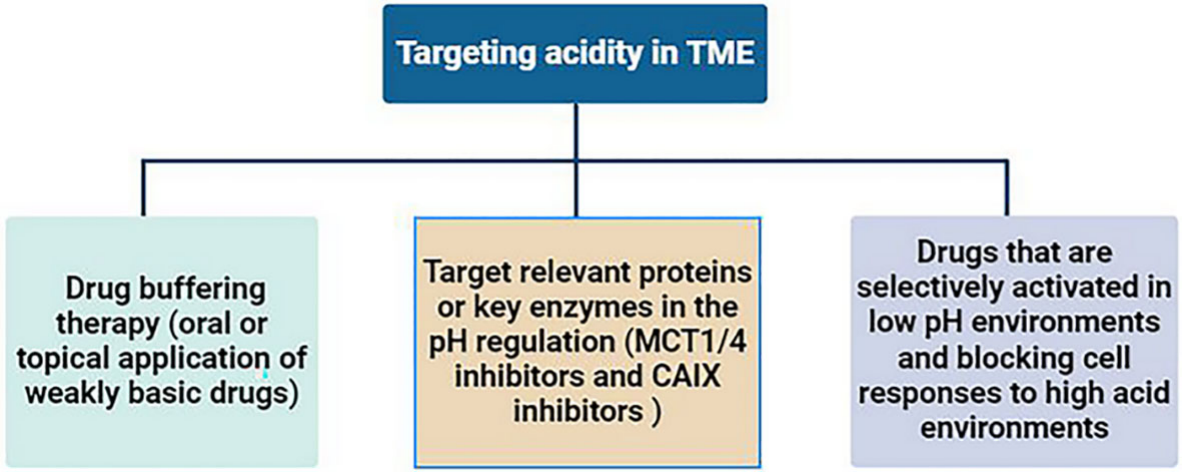
Strategies for targeting acidity in TME.

The most direct way to target the tumor’s acidic microenvironment is to neutralize the acidic components with alkaline drugs, known as drug buffering therapy. Studies have shown that oral or topical application of weakly basic drugs (such as sodium bicarbonate and calcium hydride, etc.) can neutralize the acidic environment in tumors, improve the efficacy of anti-tumor drugs and reverse immunosuppression (201–203). Target relevant proteins or key enzymes in the pH regulation mechanism within tumor cells to reduce the production of acidic products. For example, the monocarboxylate-H^+^ cotransporter 1/4 (MCT1/4) can transport lactic acid and H^+^ ions, and its selective inhibitors are still in the development stage (204). Carbonic anhydrase 9(CAIX) is also a key enzyme in the production of extracellular acidic microenvironment, mainly expressed in tumors, and plays an important role in catalyzing the hydration of CO_2_ (205). CAIX has been shown to be a predictor of poor prognosis in tumor patients (206). CAIX inhibitors are also undergoing ongoing preclinical studies (207). In addition, developing drugs that are selectively activated in low pH environments and blocking cell responses to high acid environments are also potential strategies for targeting tumor acidic microenvironments (16, 208) (**Figure 4**).

#### 4.1.3 Targeting HIFP

As mentioned above, HIFP is mainly caused by tumor hypoperfusion and increased leakage due to non-functioning blood vessels and lymphatics within the tumor, deposition of ECM components, and interstitial fibrosis. Therefore, the strategy of targeting HIFP is mainly based on regulating the vasculature and ECM within the tumor, reducing HIFP, and promoting the penetration of anti-tumor drugs into the tumor. Corresponding regulatory strategies are described in the section targeting the tumor mechanical microenvironment below.

### 4.2 Targeting the mechanical microenvironment

#### 4.2.1 Targeting CAFs

CAFs specific surface markers are potential therapeutic targets against CAFs. Since FAP is highly specifically expressed on CAFs in the stroma of most malignant tumors (more than 90% of tumors), FAP^+^ CAFs can be cleared by immunotoxins targeting FAP (95). Other approaches to target FAP include DNA vaccines and chimeric antigen receptor (CAR) T cells (209, 210). Other therapeutic targets found for CAFs include α-SMA, vitamin D receptor, PDGFE, and GPR77 (13, 211). It has been found that both FAP and α-SMA can be expressed in other cells, for example, pluripotent bone marrow stromal cells also express FAP and can be killed by drugs targeting FAP, leading to potential side effects (212). Therefore, it is necessary to conduct large-scale clinical trials to evaluate the clinical effects of anti-CAFS drugs and to find more specific CAFs targets.

While the clearance of CAFs enhances the penetration of anti-tumor drugs to tumors, the clearance of a large number of interstitial components may also disrupt the homeostasis of TME and increase the risk of tumor metastasis. Therefore, silencing CAFs, that is, targeting CAF_S_-secreted cytokines (such as IL-6, PDGF, etc.) and chemokines (CXCL12), is also an effective strategy. For example, CXCL12 plays a key role in local immunosuppression by blocking T cell infiltration; CXCR4 inhibitors (AMD3100) block the interaction between CXCL12 and CXCR4 and restoring anti-tumor immunity by enhancing tumor infiltration by CD8^+^ T cells (213). Saha et al. showed that gold nanoparticles can down-regulate TGF-β, PDGF, and basic fibroblast growth factor (bFGF) secreted by pancreatic CAFs, reshape the TME and inhibit tumor growth (214).

#### 4.2.2 Targeting ECM

ECM is an important mechanical barrier to tumors, which prevents the penetration of anti-tumor drugs into tumor tissues. Therefore, down-regulating the expression of ECM components or degrading the generated components will improve the penetration of the drug. At present, the strategies for destroying ECM can be roughly divided into the following three categories: physical methods (such as hyperthermia and high-intensity focused ultrasound, etc.) (215, 216); Biochemical enzymes (such as hyaluronidase and collagenase) (217, 218); Chemical drugs (such as cyclopamine and digoxin) (219, 220). In addition, considering the purpose of limiting tumor metastasis, some researchers use artificial materials to enhance ECM (221). For example, a laminin-mimicking polypeptide (BP-KLVFFKGGDGR-YIGSR) can bind integrin and laminin receptors within tumors and inhibit lung metastasis in melanoma and breast cancer models (222).

Matrix-Stiffening Biomaterials aiming at increasing the mechanical stiffness of the ECM can restrict tumor cell movement, but their dense structure may limit nanoparticle diffusion, and engineering these materials with degradable components or optimal porosity may mitigate this issue. Drugs, such as LOXL2 (lysyl oxidase-like 2) Inhibitors, aiming at reducing ECM crosslinking, can lead to a softer, more permeable matrix. By softening the ECM, they enhance nanoparticle penetration, making them advantageous for drug delivery. However, the potential risk of increased tumor cell mobility must be carefully managed. Both the above strategies have unique advantages and challenges in modulating the ECM for tumor containment and nanoparticle delivery. Combining these approaches with careful tuning of ECM properties may offer a promising strategy to improve cancer therapy outcomes. For example, transient LOXL2 inhibition could soften the ECM to enhance nanoparticle penetration, followed by matrix-stiffening biomaterials to physically contain the tumor.

#### 4.2.3 Targeting angiogenesis

The therapeutic strategy of anti-tumor angiogenesis is to target proteins associated with the angiogenesis pathway (such as VEGF/VEGFR, etc.). At present, the FDA has approved a variety of monoclonal antibodies targeting angiogenesis signaling pathways (such as bevacizumab and cetuximab, etc.) and tyrosine kinase inhibitors (sorafenib and lenvatinib, etc.) for the treatment of tumors, and many preclinical studies have demonstrated their significant anti-tumorigenesis effects (223). However, in clinical practice, anti-angiogenesis therapy can only play a short-term effect in a small range of specific tumor populations, and large doses or long-term use of anti-angiogenesis drugs often lead to excessive shrinkage of tumor blood vessels, increased vascular invasiveness, and drug resistance. This is mainly related to its exacerbation of tumor hypoxia, the upregulation of other alternative angiogenic factors (i.e., anti-angiogenic drugs cannot cover all target proteins associated with angiogenesis), and the remodeling of TME (108). Besides, researchers have confirmed that anti-VM therapeutic agents could inhibit the tumor angiogenesis, and a combination of VM-targeting and endothelium-targeting anti-angiogenic drugs could exert greater anti-angiogenesis effect and inhibit the growth of tumors more efficiently than each agent alone (89, 224, 225).

### 4.3 Targeting the metabolic microenvironment

Currently, drugs targeting tumor metabolism mainly target transporters and key enzymes in the core metabolic pathways of tumor cells, and these drugs have shown promising therapeutic value in preclinical/clinical trials. Glucose metabolic targets mainly include glucose transporters, hexokinase-2, glyceraldehyde 3-phosphate dehydrogenase, monocarboxylic acid transporters, carbonic anhydrase 9/12, mTORC1/mTORC2 pathway, PI3K/mTOR pathway, KRAS mutations, and et al. (226). The targets targeting glutamine metabolism mainly include glutaminase, glutamine synthetase, and glutamine cell entrance transporter SLC1A5 (227). The targets targeting lipid metabolism include the mevalonate pathway, hydroxymethylglutaryl CoA reductase, and squalene cyclooxygenase (228). Other reported methods for targeting metabolism include targeting polyamine metabolism, serine metabolism, forkhead transcription factor family (FOXOs), and et al. (229).

Recent studies have indeed highlighted the role of fatty acid oxidation (FAO) in the survival and function of regulatory T cells (Tregs) within TME. Tregs rely on FAO for their metabolic needs, and inhibiting this pathway could potentially destabilize Tregs, thereby enhancing anti-tumor immunity (230). CPT1A (carnitine palmitoyltransferase 1A) is a key enzyme in FAO, facilitating the transport of fatty acids into the mitochondria for oxidation. CPT1A inhibition can destabilize these immunosuppressive cells, thereby enhancing the efficacy of PD-1 blockade and promoting a more robust anti-tumor immune response (231–235). However, current studies are less, and further clinical studies are needed to validate these findings and explore the potential of this combination therapy in human patients.

Microbes also play an important role in regulating tumor metabolism. At present, the main strategies for targeting microorganisms include fecal microbial transplantation, targeting microorganisms using single strains or engineered synthetic strains, diet and probiotic regulation, antibiotics targeting a certain flora, and phage-based pathways (139, 149, 236). Fecal Microbiota Transplantation (FMT) is an emerging therapeutic approach that involves transferring fecal matter from a donor to a recipient to restore a balanced gut microbiota. Several clinical trials have explored how modulating the gut microbiome through FMT could influence cancer treatment outcomes, including response to immunotherapy, chemotherapy, and management of cancer-related complications, the early results are encouraging (237–241). However, more robust clinical trials are needed to establish its safety, efficacy, and optimal application in oncology. The challenges in standardizing microbiome-based therapies include interindividual variability, lack of standardization in FMT, safety concerns, regulatory and ethical issues, mechanistic complexity, and Production availability (242). As the field advances, FMT could become a valuable component of personalized cancer therapy.

Although some progress has been made in targeting tumor metabolism, significant challenges remain. First, drugs that specifically target tumor cell metabolism are still lacking, and the use of drugs that target metabolism may damage normal cells. For example, the survival of immune cells is also dependent on glycolysis and glutamine metabolism, so targeting related metabolic pathways induces an immunosuppressive response (243). Secondly, tumor cell metabolism and stromal cell metabolism should be considered as a whole, and they influence each other in TME. Therefore, it is necessary to find highly specific targets for tumor cell metabolism and observe the long-term efficacy and side effects of targeted drugs.

### 4.4 Targeting the inflammatory microenvironment

Clinically, the use of the anti-inflammatory drug, aspirin, can inhibit tumor survival and metastasis, suggesting that targeting tumor-associated inflammation is a potential therapeutic target for anti-tumor treatment (244). The main strategy for targeting tumor-related inflammation is to target the cells involved in the inflammatory process and inflammatory mediators (including cytokines and chemokines).

As mentioned above, TAMs are major inflammatory cell populations that secrete a variety of inflammatory mediators, such as CCL2 and CXCL8, and maintain tumor proliferation and immunosuppressive phenotype by inducing the transformation of TAMs from pro-inflammatory M1 type to anti-inflammatory M2 type. Therefore, the approach to targeting TAMs is an important target for tumor-associated inflammation. Toll-like receptor agonists (R848) can activate toll-like receptors and promote the transformation of TAMs to the M1 phenotype, showing anti-tumor efficacy in mouse tumor models (245). The combination of CD40 and colony-stimulating factor 1 receptor antagonists leads to the transformation of TAMs into pro-inflammatory phenotypes and induces the response of effector T cells (246). Other agents that can reprogram TAMs include histone deacetylase inhibitors (247), antibodies against macrophage collagen-containing receptors (248), PI3K-γ inhibitors (249), and anti-CD47 antibodies (250), all of which have shown good antitumor activity in preclinical studies/early clinical trials. Elimination of TAMs in tumors is also a strategy to target TAMs. Currently, the main approaches include the use of small molecules or antibodies targeting colony-stimulating factor 1 receptors (such as PLX3397, JNJ-40346527, PLX7486, etc.) and diphosphonates (251). In addition, inhibiting the recruitment of TAMs by inhibiting CCL2-CCR2 signaling has also shown effective anti-tumor effects (252).

Targeting key inflammatory mediators related to tumor inflammation, including cytokines (such as IL-1, IL-6, TNF-α, etc.) or major regulators of the inflammatory response (such as transcription factors NF-kB and STAT3, etc.), may inhibit tumor development (13). For example, a monoclonal antibody against the IL-6 receptor (Tocilizumab) enhances the antitumor activity by blocking the IL-6 receptor (253). Infliximab has demonstrated outstanding anti-tumor function in a phase 2 trial of kidney cancer by anti-TNF-α (254). TGF-β is also an important target because it is a major regulator of chronic inflammation. TGF-β inhibitors mainly include bifunctional antibodies, receptor kinase inhibitors, antisense oligonucleotides, and TGF-β-related vaccines (155, 255). However, anti-tumor studies using TGF-β inhibitors have shown conflicting results, possibly due to abnormally altered or non-functional TGF-β signaling in some tumors (256). Optimized combination therapy strategies and more clinical trials are needed to prove their efficacy.

### 4.5 Targeting the immune microenvironment

In recent years, with the emergence of new immune checkpoint drugs, the field of tumor therapy has undergone great changes, and immunotherapy has been paid more and more attention by researchers and clinicians. PD-1 and anti-cytotoxic T lymphocyte-associated antigen 4 (CTLA-4) are the most studied immune checkpoints, both of which are expressed on the surface of T cells and when activated by binding with their ligands, inhibit the activity of T cells (257). As a result, immune checkpoint inhibitors have gradually been developed to block their binding. At present, there are many immune checkpoint inhibitors, such as Nivolumab, Pembrolizumab, Atezolizumab, and Durvalumab, which inhibit PD1/L1, and Ipilimumab and Tremelimumab, which inhibit CTLA-4, etc. (258). These drugs have demonstrated strong anti-tumor efficacy in preclinical studies/clinical studies.

**Figure 5:**
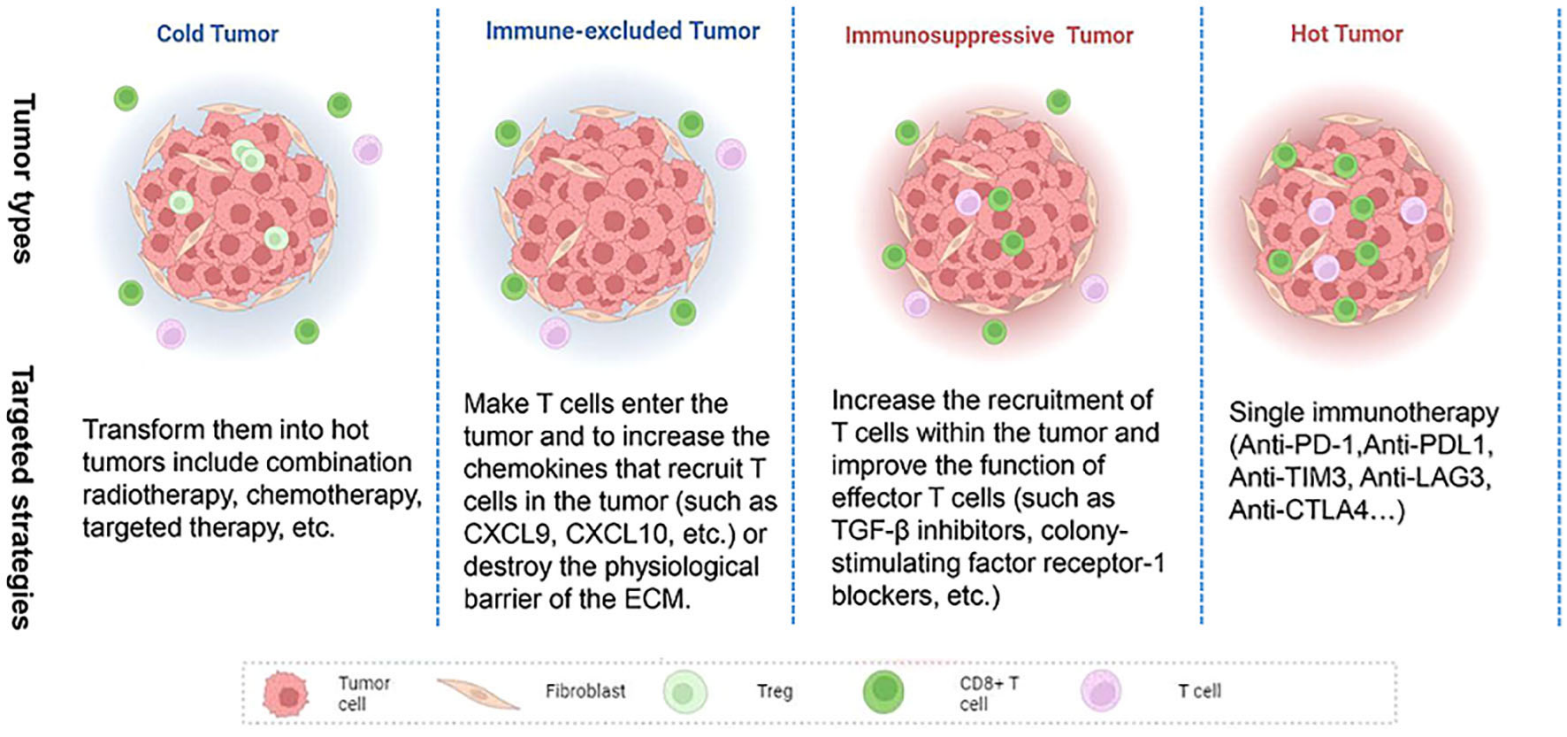
Targeted strategies for different immune-type tumors.

However, clinically, the efficacy of immune checkpoint inhibitors only works in a subset of the population. Related studies have found that the prognosis is related to the nature of the individual tumor, that is, as we mentioned above, hot tumors tend to respond to immunotherapy, while cold tumors and immunosuppressed/excluded tumors do not respond or respond weakly to immunotherapy; Pre-existing immunity (usually T cells) at the tumor site is a key condition for effective immunotherapy (152). Wang Lianjie et al. stated that the hot and cold tumor is similar to the “Yin and Yang” attributes in traditional Chinese medicine theory. The “hot and cold tumor” was believed to be a relative, but also a mutual root, and they can be transformed, normalized, and keep a dynamic balance. The above theory sets the foundation for transforming the cold tumor into a hot tumor, which provides a promising direction for targeting the cold tumor (259). Several methods aimed at improving the immune microenvironment of tumors (transforming tumors into hot tumors) are already being investigated. For immune-excluded tumors (CD8^+^ T cells are located only at the tumor edge), the therapeutic goal is to make T cells enter the tumor and to increase the chemokines that recruit T cells in the tumor (such as CXCL9, CXCL10, etc.) or destroy the physiological barrier of the ECM. Reported therapeutic strategies include lymphotoxin β-receptor signaling agonists (TNFSF14), activating β-catenin pathways, and CD73 blockers (260–262). For immunosuppressive tumors (with a small amount of T cell infiltration within the tumor), the main objective of treatment is to increase the recruitment of T cells within the tumor and improve the function of effector T cells. Reported therapeutic strategies include TGF-β inhibitors, colony-stimulating factor receptor-1 blockers, and selective targeting of γ subtypes of phosphatidylinositol 3-kinase (263–265). For cold tumors (without T cell infiltration in and around the tumor margins), currently reported methods to transform them into hot tumors include combination radiotherapy, chemotherapy, targeted therapy, etc. The type, timing, and dose of combination therapy depend on further research in the future (266–268) (**Figure 5**).

Stimulator of Interferon Genes (STING) is activated by cyclic dinucleotides (CDNs), which are produced by cyclic GMP-AMP synthase (cGAS) in response to cytosolic DNA. Upon activation, STING translocates from the endoplasmic reticulum to the Golgi apparatus, where it recruits and activates TANK-binding kinase 1 (TBK1). TBK1 then phosphorylates the transcription factor IRF3, leading to the production of type I interferons and other cytokines. This pathway is essential for the immune response to viral and bacterial infections, as well as for anti-tumor immunity (269). STING agonists are a class of compounds that activate the STING pathway. Ongoing study aims to develop more potent and selective STING agonists, improve delivery methods, and better understand the downstream effects of STING activation to maximize therapeutic benefits while minimizing adverse effects, and explore the effects of STING agonists in combination with other therapies (270, 271).

Oncolytic viruses are a type of virus that selectively infects and kills tumor cells while sparing normal, healthy cells. These viruses can be naturally occurring or genetically modified to enhance their cancer-targeting abilities (272). Oncolytic virus therapy is an emerging form of immunotherapy that leverages the virus’s ability to destroy tumor cells by selective infection replication and cell lysis, and activate the immune system to fight cancer. Reported oncolytic viruses included Talimogene laherparepvec (T-VEC), Adenoviruses, Reovirus, and Measles Virus (273). Research is ongoing to improve the specificity, efficacy, and safety of oncolytic viruses. This includes engineering viruses to express additional therapeutic genes, combining them with other immunotherapies, and developing novel delivery methods, and clinical trials are exploring their use in various cancers, including glioblastoma, pancreatic cancer, and breast cancer (272, 274, 275).

## 5 Summary and outlook

TME has been shown to play a key role in the occurrence, development, metastasis, and drug resistance of tumors. The current research results show that the components of TME interact with each other to form a complex and staggered relationship network. Although many components of TME are well known, the characteristics of each component and their interrelationships remain to be understood. Existing techniques for characterizing the TME are still lacking. First, these techniques are still only used in cell, animal tests and pre-clinical study, and their clinical application needs further investigation. Secondly, TME is a dynamic process, the existing technology cannot be real-time accurate dynamic observation. Finally, limited by the existing technical conditions and cognition, there may be undiscovered TME components and their signaling relationships.

Many TME targets have been successful in preclinical/clinical studies, suggesting that TME-targeted therapy is a promising anti-tumor strategy. However, there are still significant challenges in targeting TME therapies. First, the characteristics of the same type of tumor are different in different populations, so it is necessary to comprehensively evaluate the characteristics of individual tumors to target TME. Secondly, due to the interaction and signal crossing among various components in TME, single-target drugs may not achieve the ideal anti-tumor effect, and the development of multi-target drugs or combination drugs seems to solve this problem. In addition, since the experimental animal tumor model cannot accurately simulate the human tumor TME, future research should focus on developing models that are more consistent with the characteristics of human tumors. Finally, current drugs targeting TME may damage normal cells while targeting TME components, so it is necessary to find more specific targets.

In conclusion, this review summarizes the characteristics of TME in solid tumors and its influence on tumor occurrence, development, and metastasis, summarizes the existing methods for detecting TME, and summarizes the current strategies and potential therapeutic targets for TME. TME is composed of a variety of complex components, and the characteristics of each component and their interrelationships remain to be deeply understood. Existing techniques for characterizing the TME are still lacking. Many TME targets have been successful in preclinical/clinical studies, but there are still significant challenges for therapies targeting TME. In the future, the development of models more in line with the characteristics of human tumors, the development of new devices that can accurately detect TME, the development of high-specific targets or multi-target therapeutic drugs, or the combination of therapy with targeted tumor cells is expected to further improve the therapeutic effect of tumors.
